# The clinical impact of recent amphetamine exposure in aneurysmal subarachnoid hemorrhage patients

**DOI:** 10.3389/fneur.2024.1480401

**Published:** 2025-01-07

**Authors:** Jeffrey R. Vitt, Roger C. Cheng, Jason Chung, Travis Caton, Bo Zhou, Nerissa Ko, Karl Meisel, Edilberto Amorim

**Affiliations:** ^1^Departments of Neurologic Surgery and Neurology, University of California, Davis, Sacramento, CA, United States; ^2^Department of Neurology, Rutgers – Robert Wood Johnson Medical School, New Brunswick, NJ, United States; ^3^Department of Neurosurgery, University of California, San Francisco, San Francisco, CA, United States; ^4^Department of Radiology, University of California, San Francisco, San Francisco, CA, United States; ^5^Department of Neurology, Weill Institute for Neurosciences, University of California, San Francisco, San Francisco, CA, United States

**Keywords:** aneurysmal subarachnoid hemorrhage, transcranial doppler ultrasound, cerebral angiography, amphetamines, vasospasm

## Abstract

**Background:**

Amphetamines possess sympathomimetic properties that can affect cerebral vasculature though conflicting reports exist about their effect on vasospasm risk and clinical outcomes in aneurysmal subarachnoid hemorrhage. This study aimed to characterize the impact of recent amphetamine use on vasospasm development in aneurysmal subarachnoid hemorrhage as well as neurological outcomes.

**Methods:**

We retrospectively screened 441 consecutive patients admitted with a diagnosis of subarachnoid hemorrhage who underwent at least one cerebral digital subtraction angiogram. Patients were excluded if no urinary toxicology screen was performed within 24 h of admission, if there was a diagnosis of non-aneurysmal subarachnoid hemorrhage, or if ictus was greater than 72 h from hospital admission. Vasospasm characteristics were collected from digital subtraction angiography and transcranial Doppler studies.

**Results:**

129 patients were included and 24 tested positive for amphetamines on urine drug screen. No significant differences were found in respect to patient age, sex, or admission clinical severity scales (Hunt-Hess and modified Fisher) based on amphetamine use. There was no difference in the severity of vasospasm or time to peak severity based on recent amphetamine use. A trend toward more isolated posterior circulation vasospasm was observed in the amphetamine present group (16.7% vs. 4.8%, *p* = 0.06), while there was higher incidence of anterior circulation vasospasm in the amphetamine absent group (79.2% vs. 94.3%, *p* = 0.03). There was no difference in delayed cerebral ischemia incidence, length of hospital stay, need for ventriculoperitoneal shunt placement, functional outcome at discharge or hospital mortality based on amphetamine use.

**Interpretation:**

Recent amphetamine use was not associated with worse vasospasm severity or delayed cerebral ischemia in aneurysmal subarachnoid hemorrhage patients. Further investigations about localized effects in the posterior circulation and impact on long-term functional outcomes are warranted.

## Introduction

Aneurysmal subarachnoid hemorrhage (SAH) accounts for approximately 5% of strokes with an annual incidence of approximately 6–10 per 100,000 persons (1, 2). While the overall incidence of SAH has been decreasing globally over the past several decades, SAH carries a high burden of mortality with a case fatality rate of 27–44% (2–5). Nearly half of SAH survivors will not achieve functional independence at one year and many continue to experience significant long-term deficits in memory, executive function, and language (3, 6).

Certain recreational substances such as tobacco, cocaine, cannabis, and amphetamine compounds have been tied to the development of vasospasm and heightened risk of delayed cerebral ischemia (DCI) after SAH (7–10). Approximately 30% of SAH patients develop DCI, a diagnosis strongly associated with long-term neurologic disability and mortality (11–13). The underlying pathophysiology of DCI is likely multifactorial, and includes microcirculatory disfunction, loss of autoregulation, cortical spreading depolarizations, metabolic mismatch, and microthrombi formation leading to cerebral hypoperfusion (14). Large vessel vasospasm is a common finding in SAH patients and is also a potential contributor to DCI by impairing cerebral blood flow (CBF) and vascular adaptation to metabolic demand (12, 15). Psychoactive drugs can exert direct vascular alterations in cerebral arteries, but the pathways activated by these drugs contributing to DCI after SAH have not been elucidated (7, 16, 17).

Amphetamine-type stimulants (AMP) including methamphetamine are becoming increasingly more common drugs of abuse and exert potent sympathomimetic properties (18, 19). Chronic exposure has been associated with a variety of cerebrovascular complications, including increased risk for aneurysm formation and rupture (17, 20, 21). Contradicting reports exist however on the impact of AMP use on the development of vasospasm and DCI in SAH patients, however these studies did not delineate differences between chronic and recent AMP exposure (8, 22, 23). In this study, we aimed to characterize the influence of recent AMP use, as evident by positive urine drug test on admission, on the time course and severity of vasospasm as well as DCI occurrence following SAH leveraging multimodal imaging.

## Methods

### Patient population

Electronic health records (EHR) were reviewed between December 28, 2011 and January 1, 2019 and for all subjects admitted to the University of California San Francisco (UCSF) Medical Center with a diagnosis non-traumatic SAH based on ICD codes (430 and 160.9) and at least one digital subtraction angiography (DSA) performed. Subjects were excluded if they were younger than 18 years of age, did not have an aneurysmal source for SAH, did not have a urine toxicology screen within 24 h of admission, and if time of aneurysmal rupture was greater than 72 h from admission.

### Ethics and patient consent

This study was approved under the UCSF Institutional Review Board. Need for informed consent was waived for analysis of data obtained as part of routine medical care.

### Clinical data

Demographics including age, sex, admission status (direct from emergency department versus transfer from outside facility), onset of SAH, aneurysm location and urine toxicology results were collected from EHR. If subjects tested positive for AMP on urine toxicology screening on admission, they were included in the AMP cohort. Hunt and Hess (HH) classification was abstracted from the admission documentation and dichotomized into high (scores 4 and 5) and low (scores 1–3). The modified Fisher Scale (mFS) was determined from admission head computed tomography (CT) and dichotomized into high (score of 4) or low (scores 1–3). Information on external ventricular drain (EVD) placement, duration of drainage as well as if a ventriculoperitoneal shunt (VPS) placement was abstracted. Hospital and intensive care unit (ICU) length of stay as well as discharge disposition (home, acute rehabilitation, skilled nursing facility, acute care facility, hospital transfer or death) and modified Rankin Scale (mRS) at time of discharge were recorded. A good functional outcome was considered as a mRS ranging 0–3 (i.e., no symptoms to at least able to walk unassisted).

Delayed cerebral ischemia was abstracted from the chart and defined as either a new focal neurologic impairment or decrease of at least 2 points on the Glasgow Coma Scale lasting for at least one hour and was not immediately apparent after aneurysm occlusion nor could be attributed to another cause (24). Delayed infarction on either CT or magnetic resonance imaging (MRI) not attributable to surgical procedures or endovascular treatment was also considered evidence of DCI.

Severity of vasospasm on DSA was scored by dual board-certified Neuroradiology and Neurointerventional Radiology faculty and rated as absent, mild, moderate or severe using previously published criteria (25). Subjects underwent at least one DSA and may have had repeat angiograms as clinically indicated. Transcranial Doppler (TCD) assessment is routinely pursued for vasospasm surveillance in our institution, with results interpreted by an attending Vascular Neurologist with certification in Neurosonology. For statistical analysis, vasospasm was considered anterior predominant or posterior predominant depending on where the most severe vasospasm was recorded on either DSA or TCD. In cases where the severity of vasospasm was equal in the anterior and posterior circulation the vasospasm was considered multifocal.

### Transcranial Doppler testing

TCD was performed as clinically indicated by a Registered Vascular Technologist with specialization in TCD using a 2-MHz probe and ST3 Transcranial Doppler (Spencer Technologies, Redmond, WA, USA). Vasospasm was classified as mild, moderate and severe by mean flow velocities (MFV) of 120–139 cm/s, 140–179 cm/s, and > 180 cm/s with corresponding Lindegaard ratios of 3.0–3.99, 4.0–5.99, and > 6.0 in the anterior circulation vessels and basilar/vertebral artery ratios (MFV Basilar Artery)/(Right Vertebral Artery + Left Vertebral Artery)/2 of 2.7–3.29, 3.3–4.3, and > 4.3 in the posterior circulation. When disagreement between MFV and ratio measurements was identified, final determination was made according to whichever value corresponded to the lower overall severity.

### Statistical analysis

Continuous variables and categorical variables are reported as medians with interquartile ranges and frequencies with percentages. We utilized the Shapiro–Wilk test to evaluate normality distribution. Chi-square was used to compare categorical data. The Student’s t-test was used for variables with normal distribution and Mann–Whitney for non-normally distributed data. Logistic regression analysis was used to compare the incidence of DCI, occurrence of severe vasospasm only (i.e., binary), and time from SAH onset to peak vasospasm using both DSA and TCD based on AMP use. The following risk factors for vasospasm were assessed using univariable logistic regression: age, positive AMP urine test, HH and mFS. Variables with a *p* of ≤0.1 in the univariable analysis were included in the multivariable logistic regression model for DCI and time to peak vasospasm. For assessment of vasospasm of any severity (i.e., all severity levels included), an ordinal logistic regression analysis was performed using a proportional odds logistic regression (POLR) analysis with the variables positive AMP urine test, age, HH and mFS. A backward stepwise logistic regression using Akaike Information Criterion (AIC) was pursued for feature selection. A final POLR analysis was then employed to identify the variables most strongly associated with vasospasm severity. Ordinal regression was performed using the MASS package (26). Separate analyses were performed using vasospasm severity defined by TCD or DSA criteria. Data were analyzed using R version 3.5.1 (R Foundation for Statistical Computing, Vienna, Austria).

## Results

**Figure 1:**
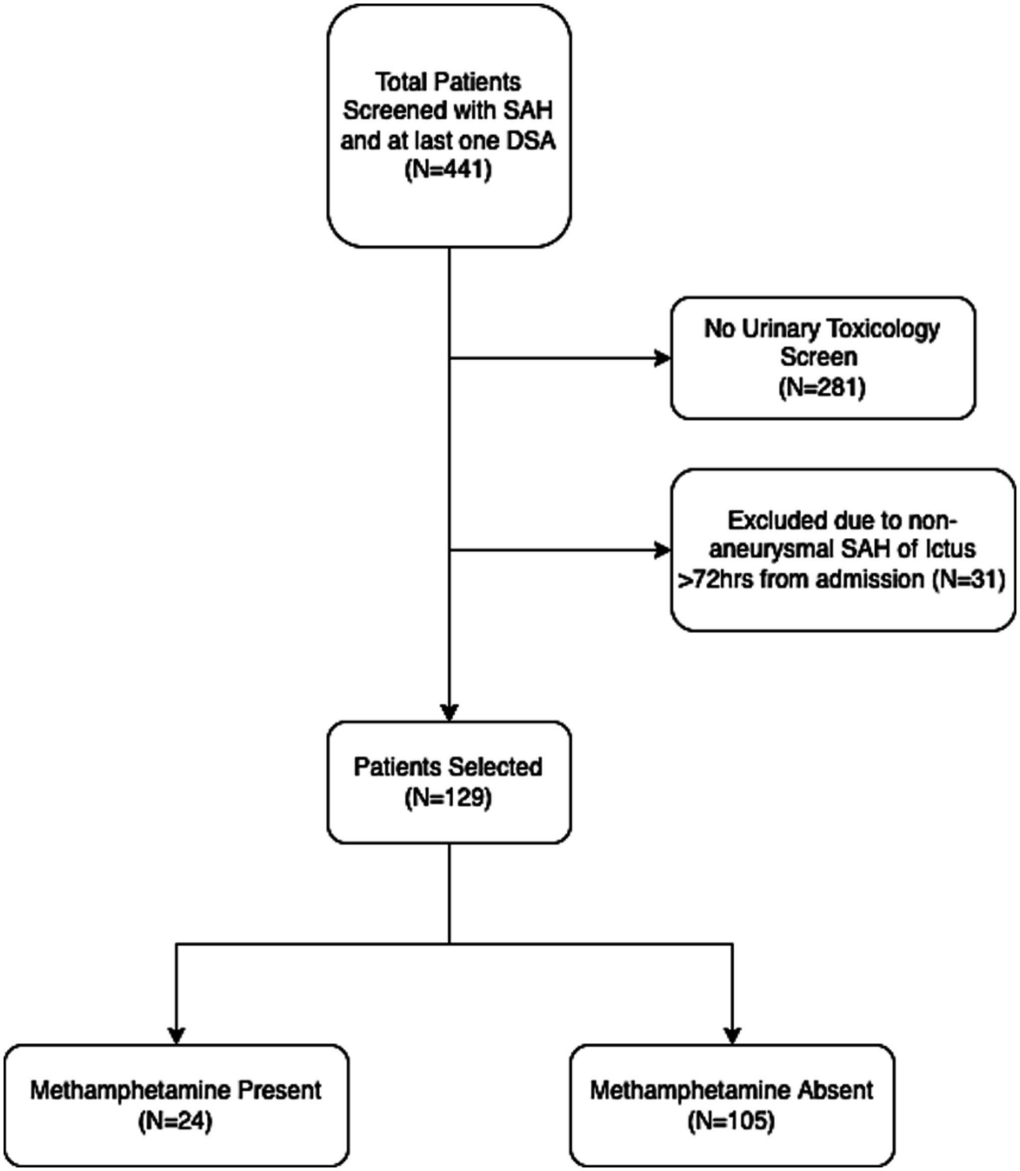
Flow chart of patient selection. SAH, subarachnoid hemorrhage; DSA, digital subtraction angiography.

A total of 441 subjects with a primary diagnosis of non-traumatic SAH and underwent at least one DSA were screened. Of these, 281 subjects were excluded due to lack of urinary toxicology screen performed within 24 h of admission. An additional 31 subjects were excluded due to non-aneurysmal source of hemorrhage or ictus greater than 72 h from admission. The remaining 129 subjects were included for primary analysis, including 24 subjects in the AMP present group (Figure 1). There was no significant difference in AMP present and AMP absent groups regarding age (50.1 vs. 51.4, *p* = 0.62), female sex (66.7% vs. 63.8%, *p* = 0.87), admission HH (median 3 for both groups), or mFS (median 4 for both groups). Most subjects were transferred from an outside facility (95.8% vs. 87.6%, *p* = 0.42). There was no difference in aneurysm location or rate of EVD insertion (Table 1).

**Table 1:** Demographics of amphetamine absent and amphetamine present cohorts.

	Amphetamine absent (*N* = 105)	Amphetamine present (*N* = 24)	*p*-value
Age [Mean, SD]	51 (11.4)	50 (11)	0.62
Female (%)	67 (63.8%)	16 (66.7%)	0.87
Transfer from Outside Facility (%)	92 (87.6%)	23 (95.8%)	0.42
Aneurysm location (%)			
Anterior circulation	84 (80%)	17 (70.8%)	0.48
Posterior circulation	21 (20%)	7 (29.2%)	
Modified Fisher Scale [Median, IQR]	4.00 (2.00, 4.00)	4.00 (3.00, 4.00)	0.36
Hunt and Hess [Median, IQR]	3.00 (2.00, 4.00)	3.00 (3.00, 4.00)	0.07
EVD Inserted (%)	65 (61.9%)	18 (75%)	0.33

EVD, external ventricular drain; SD, standard deviation; IQR, Interquartile range.

**Figure 2:**
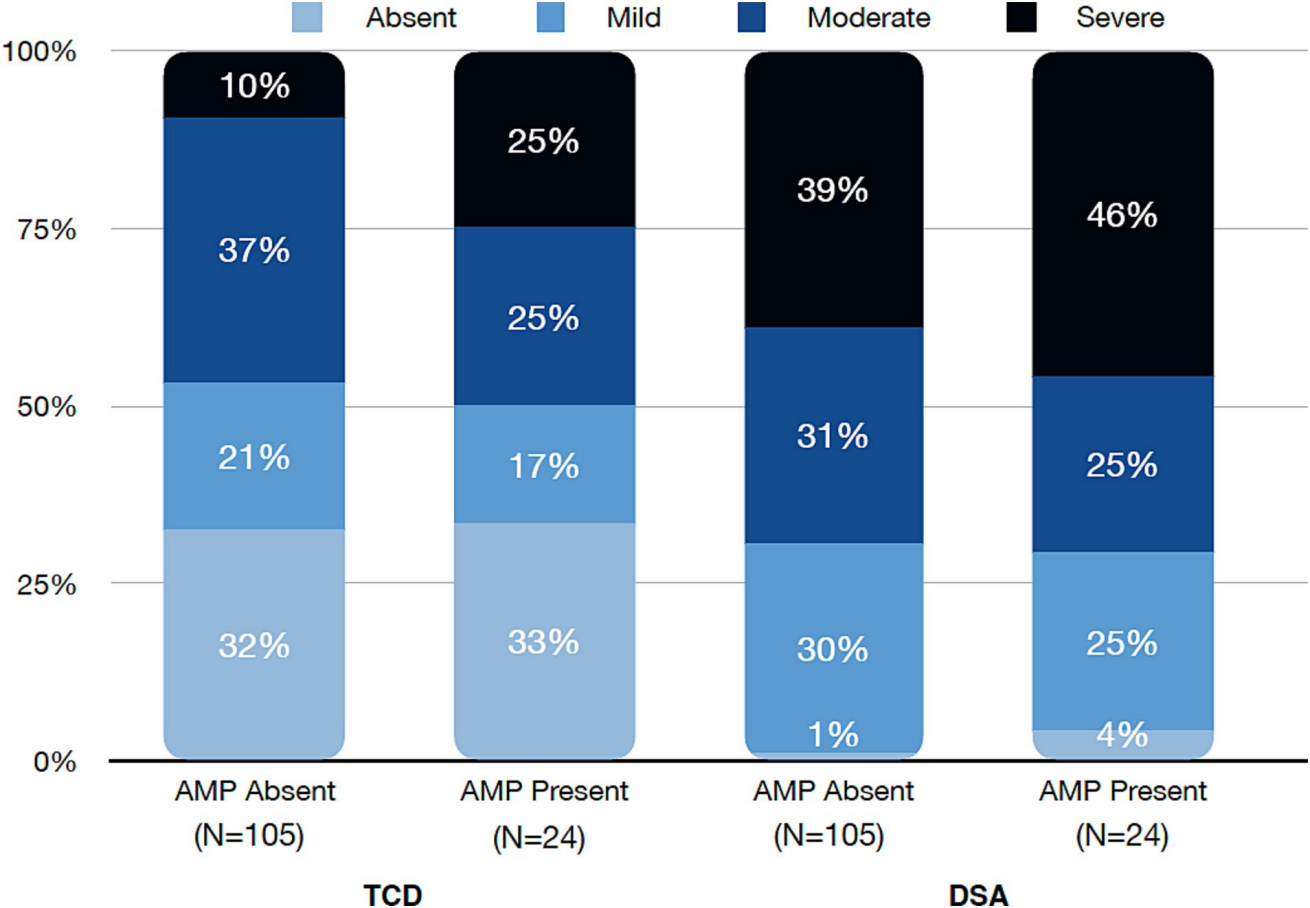
Severity grade of vasospasm by Transcranial Doppler (TCD) and digital subtraction angiography (DSA) divided into amphetamine (AMP) absent or present cohorts.

While vasospasm of any grade was more commonly diagnosed with DSA than TCD, there was no difference in vasospasm incidence for the AMP present or AMP absent groups for either modality (95.8% vs. 99%, *p* = 0.82) and (66.7% vs. 67.6%, *p* = 1), respectively. When evaluating only cases of severe vasospasm, similarly there was no statistical difference in respect to AMP use with DSA (45.8% vs. 39%, *p* = 0.7) or TCD (25% vs. 9.5%, *p* = 0.08) (Figure 2). Time to peak vasospasm was similar in both groups on DSA (6.5 vs. 7 days, *p* = 0.32) or TCD (5.5 vs. 6 days, *p* = 0.95) (Table 2). There was also no notable difference in the incidence of DCI between the two groups (20.8% vs. 21.9%, *p* = 1). Both groups underwent a similar amount of DSA procedures (median 3.5 vs. 3, *p* = 0.37) throughout the hospital stay and each group had a median of 5 TCD examinations performed. Vasospasm of any severity involving the anterior circulation was more commonly observed in the AMP absent group (79.2% vs. 94.3%, *p* = 0.03) and there was a higher proportion of isolated posterior circulation vasospasm in the AMP present group (16.7% vs. 4.8%, *p* = 0.06), though this did not reach statistical significance.

**Table 2:** Clinical outcomes for amphetamine absent and amphetamine present cohorts.

	Amphetamine absent (*N* = 105)	Amphetamine present (*N* = 24)	*p*-value
Delayed cerebral ischemia (%)	23 (21.9%)	5 (20.8%)	1
Any vasospasm present (%)			
TCD	71 (67.6%)	16 (66.7%)	1
DSA	104 (99%)	23 (95.8%)	0.82
Severe vasospasm present (%)			
TCD	10 (9.5%)	6 (25.0%)	0.08
DSA	41 (39%)	11 (45.8%)	0.7
Time to peak vasospasm (Days) [Median, IQR]			
TCD	6 [4, 9.5]	5.5 [3, 10]	0.84
DSA	7 [5, 9]	6.5 [4, 9]	0.33
Location of peak vasospasm (%)			0.08
Anterior	75 (71.5%)	16 (66.7%)	
Posterior	5 (4.8%)	4 (16.7%)	
Multifocal	24 (22.9%)	3 (12.5%)	
Number of TCD Performed [Median, IQR]	5 [3, 7]	5 [3, 6]	0.92
Number of DSA Performed [Median, IQR]	3 [2, 4]	3.5 [2, 4]	0.37
Duration of EVD (Days) [Median, IQR]	18 [15, 20]	17 [15, 21]	0.63

TCD, transcranial Doppler; DSA, digital subtraction angiography; EVD, external ventricular drain; VPS, ventriculoperitoneal shunt; ICU, intensive care unit; mRS, Modified Rankin Scale; IQR, Interquartile range.

Ordinal regression analysis revealed no influence of AMP use on vasospasm severity, while younger age and higher mFS were found to be associated with increased risk for both TCD and DSA (Table 3). When examining incidence of severe vasospasm exclusively, there was an increased risk associated with AMP exposure using TCD for diagnosis in univariate analysis, however this association was no longer significant in multivariable analysis. This association was not present when evaluating vasospasm by DSA (Table 4). Likewise, AMP use was not associated with development of DCI and did not influence the time course of vasospasm by either TCD or DSA criteria in univariate or multivariable analysis. In contrast, younger age as well as higher mFS and HH on admission were associated with increased risk of severe vasospasm development diagnosed by DSA. With respect to DCI, univariate analysis revealed an association with higher HH, mFS, and severe vasospasm diagnosed with TCD or DSA, though with multivariable analysis, only admission HH and severe vasospasm diagnosed on DSA were found to be associated with DCI occurrence.

**Table 3:** Ordinal logistic regression analysis for predictors of vasospasm of any severity diagnosed by transcranial Doppler (TCD) and digital subtraction angiography (DSA).

	Ordinal logistic regression for vasospasm	
	OR (95% CI)	*p*-value
TCD		
Amphetamine present	--	--
Age	0.94 (0.92–0.98)	< 0.001
Hunt and Hess	--	--
Modified Fisher Scale	2.18 (1.11–4.26)	0.023
DSA		
Amphetamine present	--	--
Age	0.95 (0.93–0.99)	0.009
Hunt and Hess	1.84 (0.85–4.00)	0.124
Modified Fisher Scale	3.32 (1.62–6.80)	0.001

**Table 4:** Univariate and multivariable analysis for development of severe vasospasm and time to peak vasospasm as diagnosed by transcranial Doppler (TCD) and digital subtraction angiography (DSA) as well as delayed cerebral ischemia (DCI).

	OR (95% CI) univariate	*p*-value	OR (95% CI) multivariable	*p*-value
Severe vasospasm (TCD)				
Amphetamine present	**3.66 (1.05–12.34)**	**0.04**	3.28 (0.91–11.43)	0.06
Age	0.98 (0.94–1.03)	0.54	0.99 (0.94–1.04)	0.65
Hunt and Hess	1.64 (0.50–5.07)	0.39	1.37 (0.38–4.58)	0.62
Modified Fisher Scale	1.53 (0.48–5.94)	0.5	01.19 (0.34–4.88)	0.79
Severe vasospasm (DSA)				
Amphetamine present	1.41 (0.56–3.51)	0.46	0.99 (0.34–2.81)	0.99
Age	**0.94 (0.91–0.97)**	**0.001**	**0.93 (0.89–0.97)**	**<0.001**
Hunt and Hess	**2.5 (1.15–5.54)**	**0.02**	**2.73 (1.12–6.97)**	**0.03**
Modified Fisher Scale	**3.44 (1.58–7.97)**	**0.001**	**3.18 (1.33–8.03)**	**0.005**
Time to peak vasospasm (TCD)				
Amphetamine present	1.66 (−0.61–3.94)	0.15	1.55 (−0.76–3.87)	0.18
Age	0 (−0.08–0.08)	0.96	0.01 (−0.07–0.09)	0.83
Hunt and Hess	0.86 (−1.07–1.07)	0.38	0.62 (−1.34–2.58)	0.53
Modified Fisher Scale	1.24 (−0.74–3.23)	0.22	1.08 (−0.95–3.11)	0.29
Time to peak vasospasm (DSA)				
Amphetamine present	−0.60 (−2.47 to 1.26)	0.52	−0.54 (−2.40 to 1.33)	0.57
Age	0.05 (−0.01 to 0.11)	0.12	0.05 (−0.01 to 0.12)	0.09
Hunt and Hess	0.23 (−1.34–1.81)	0.77	0.04 (−1.54–1.62)	0.96
Modified Fisher Scale	1.13 (−0.46–2.72)	0.16	1.33 (−0.29–2.95)	0.11
Delayed cerebral ischemia				
Amphetamine present	0.94 (0.29–2.63)	0.91	0.55 (0.14–1.86)	0.36
Age	1.01 (0.97–1.05)	0.73	1.03 (0.98–1.08)	0.23
Hunt and Hess	**4.79 (2.00–11.85)**	**0.001**	**3.51 (1.33–9.53)**	**0.01**
Modified Fisher Scale	**4.82 (1.71–17.30)**	**0.01**	3.12 (0.98–12.17)	0.07
Severe vasospasm on TCD	**3.41 (1.11–10.22)**	**0.03**	2.66 (0.74–9.55)	0.13
Severe vasospasm on DSA	**4.35 (1.82–11.09)**	**0.001**	**3.49 (1.18–11.05)**	**0.03**

Bolded sections indicate variables that met statistical significance (*p*=0.05) in univariate and multivariate analysis.

Duration of EVD use was similar in both groups (17 vs. 18 days, *p* = 0.63) as well as the rate of VPS placement (25% vs. 21%, *p* = 0.93), length of ICU stay (19.5 vs. 18 days, *p* = 0.3) and hospital length of stay (23.5 vs. 20 days, *p* = 0.06). There was no difference in rates of discharge to home or acute rehab (45.8% vs. 60%, *p* = 0.3), favorable functional outcomes at discharge (41.7% vs. 55.2%, *p* = 0.31) or hospital mortality between both groups (4.2% vs. 6.7%, *p* = 1).

## Discussion

In this retrospective analysis of subjects admitted with SAH, we found that recent exposure to AMP was not associated with the development, time course or severity of cerebral vasospasm nor with the incidence of DCI. Contrary to previously published reports, our study exclusively evaluated subjects presenting with evidence of AMP present on urine toxicology screening, therefore it was better suited to assess the potential direct interaction of systemic AMP and associated metabolites with the development of vasospasm and DCI following SAH (8, 22, 23). Using a multimodal approach with TCD and DSA, we were able to thoroughly characterize the incidence, distribution, and chronicity of vasospasm, finding less frequent development of anterior circulation vasospasm and a trend toward more isolated posterior vasospasm in subjects with recent AMP use. We found no difference in functional outcomes at discharge, hospital length of stay, VPS placement, or hospital mortality. These findings indicate that active use of AMP may not be associated with increased risk of vasospasm and DCI in SAH, and that care for these patients should follow standard protocols for surveillance.

Patient’s age at the time of SAH presentation was similar between subjects with or without recent AMP exposure in our study. The median age at presentation was 55, ranging from 28 to 64 years. This is in contrast to prior published cohorts of patients using AMP who were admitted at a much younger age on presentation, with a median age generally in the fourth decade of life (8, 22, 27). As younger age is a risk factor for vasospasm, it is possible that age might have confounded this association in previous reports. Our findings may suggest that AMP use is more common in older populations compared to historical cohorts or reflect local demographics in the Northern California region. Internationally, recreational use of AMP has risen sharply across different segments of the population with production growing worldwide (18). In the United States, AMP use has increased over the past decade, particularly among individuals using other substances of abuse, and is responsible for nearly a quarter of drug-related treatment admissions (19, 28). AMP effects the central nervous system by increasing the release of serotonin, norepinephrine and dopamine into the synaptic cleft as well as inhibiting neurotransmitter degradation thereby increasing postsynaptic activity (17). Chronic recreational AMP use has wide ranging deleterious systemic effects, particularly involving the cardiovascular and cerebrovascular systems related to catecholamine toxicity, vessel inflammation, vascular remodeling and accelerated atherosclerosis (29, 30).

Given conflicting prior reports on the impact of AMP use and cerebral vasospasm following SAH, we sought to better characterize the incidence, time course and severity of vasospasm using repeated surveillance monitoring with TCD and DSA (8, 22, 23). It is well known that AMP exerts vasoconstriction properties on the cardiopulmonary vasculature and has been linked with increased mortality and morbidity (30). In animal studies, exposure to IV AMP has been shown to induce a potent and prolonged cerebral vasospasm response involving both the large and small vessels leading to diminished CBF and infarction (16, 31). Vasospasm related to SAH is a well-described clinical phenomena, occurring in around 70% of patients by day 7 post-bleed, and when severe (>50% narrowing of luminal diameter) is an established risk factor for the development of DCI, cerebral infarction and worse neurologic outcomes (7, 15, 32, 33). Molecular studies have illuminated differential expression of nitric oxide, enthothelin-1, renin-angiotensin system as well as calcium and thrombin signaling in patients with SAH associated vasospasm suggesting a multitude of diverse pathways are responsible for the clinical phenotype (34). Despite the known vasoconstrictive properties of AMP, the impact of acute or recent AMP exposure on the development and characteristics of vasospasm related to SAH has not been well studied. Prior published studies on AMP use and SAH found no overall difference in the incidence of DCI or vasospasm after adjusting for age, however these studies included subjects both with a reported history of AMP use or positive toxicology results (8, 22). In contrast, our study only evaluated subjects with AMP present on urine toxicology screen at hospital admission, thus indicating a recent exposure. This stricter approach was pursued to determine the impact of systemic AMP and metabolites on the development of vasospasm and DCI following SAH. Our results suggest that recent AMP exposure does not influence the incidence, time course or severity of SAH associated vasospasm. With ordinal logistic regression analysis we found that young age and high mFS on admission were predictors of vasospasm on DSA and TCD in keeping with prior published reports (7, 35). Likewise, we did not find any association between AMP use and DCI, however severe vasospasm on DSA and higher HH were associated with increased incidence in multivariable analysis as expected.

This study also evaluated location of vasospasm in AMP use, a feature not explored in previous reports (8, 20, 22). While anterior circulation vasospasm was the most common location for either cohort, this was more commonly observed in those without AMP exposure while the AMP present group had a trend toward higher proportion of isolated posterior circulation vasospasm (16.7% vs. 4.8%, *p* = 0.06). The basilar and vertebral arteries are known to exhibit a higher propensity toward disrupted cerebral autoregulation and alterations in vascular tone due to a more heterogenous distribution of sympathetic innervation compared to the anterior circulation (36). It is possible that this regionalized vascular pathophysiology may confer increased predilection of vasospasm in the posterior circulation in the presence of a vasoactive substance such as AMP, however, given our small sample this association requires further evaluation in studies involving a larger patient population.

Use of AMP has also been linked with increased risk of aneurysm formation as well as accelerated growth, abnormal morphological changes and rupture, likely owing to direct toxic effects on the vasculature leading to inflammation and impaired vessel wall integrity (17, 37–39). Although multiple prior studies did not find an interaction between AMP use and aneurysm location, a recently published large case series of AMP related aneurysms found that while anterior circulation aneurysms were more common overall (77%), posterior aneurysms were disproportionately more likely to present with rupture compared to anterior aneurysms (65% vs. 32%) and rupture despite smaller size (8, 20–22). These findings may be consistent with our cohort results, which although limited by small sample size, demonstrated a trend toward higher prevalence of rupture posterior circulation aneurysms in AMP present group (29.2% vs. 20%).

In our study, we found no difference in functional outcomes, discharge disposition to home or acute rehabilitation, mortality or hospital and ICU lengths of stay with AMP use. Prior studies involving the impact of AMP use in ICH subjects found an increased length of hospital stay, largely related to need for anti-hypertensive medication infusions, though not mortality or functional independence (27, 40). In contrast, Beadell et al. reported a trend toward worse functional outcomes in AMP users presenting with SAH which reached statistical significance after age matched control sub-analysis (8). Similar findings have been demonstrated in SAH patients with recent cocaine use, another common recreational stimulant, where there was no observed difference in vasospasm severity though was associated with worse clinical outcomes at discharge (41, 42). Furthermore, using a large SAH trial registry, Moon an colleagues found that after one and three years follow up, AMP users had no increased mortality though had higher rates of severe disability and functional dependence compared to controls, despite no difference in rates of clinical or radiographic vasospasm (22). These findings suggest that, for mechanisms which are currently unknown, AMP use may exert lasting impacts that hinder neurorecovery following SAH outside of the acute hospitalization.

A notable limitation of our study is that it was underpowered to detect a significant association between AMP use and severe vasospasm, increasing the likelihood of a Type II error. Secondly, due the single-center retrospective design as well as exploratory nature of this investigation, the clinical findings should not be viewed as generalizable and interpreted with caution as they do not clearly establish a causative relationship. For the AMP present cohort, we included all subjects with a positive urine toxicology screen for AMP on admission, and therefore it is possible that not all patients were exposed to recreational sympathomimetics and may instead have taken over-the-counter or prescription agents that have been previously shown to produce false-positive results on immunoassays (ex quetiapine, bupropion, and trazodone) (43). It should also be noted that the AMP positivity on urine toxicology may be present for up to five days since last exposure, or longer depending on renal function, which may have skewed outcomes in our cohort based on timing of last use (44). Furthermore, since we only included toxicology positive subjects in the AMP present group, the influence of more remote and chronic AMP use could not be determined accurately through retrospective review. We also did not take into consideration other substances, with or without concomitant use with AMP, which may have also impacted vascular characteristics and functional outcomes (19). Urine toxicology screening was not protocolized and became more routine over the course of the study however the potential for selection bias based on demographics or other patient characteristics is present and may have influenced our results. Moreover, subjects were included in this study only if they had at least one DSA performed as part of routine standard of care, what may have introduced a selection bias toward patients who were more likely to receive aggressive treatment. The use and frequency of TCD and DSA was not standardized and was left up to the clinical judgment of the treating team. While the number of DSA and TCD studies did not differ significantly between AMP present and absent groups, it is possible that vasospasm severity or high-risk clinical features, including AMP use, may have influenced the frequency of studies performed as well as the characterization of vasospasm time-course and interventions. Our designation of vasospasm severity was made by both TCD and DSA modalities, which are known to have modest interrater agreement, particularly with non-severe vasospasm and in vessels outside of the MCA (45, 46). Finally, our study only assessed patient outcome at time of discharge or transfer from the hospital and therefore was unable to assess long term impacts of AMP use on patient recovery and functional outcomes.

## Conclusion

In patients presenting with SAH, recent AMP use did not impact the incidence, chronicity or severity of vasospasm nor the occurrence of DCI. Our study supports prior established risk factors for vasospasm including young age and increased volume of hemorrhage on presentation independent of AMP exposure. These findings support the use of standard vasospasm surveillance practices in patients with recent AMP exposure presenting with SAH. The influence of recent AMP use and distribution of vasospasm with possible predilection for the posterior circulation warrants further investigation in subsequent studies.

## Author’s note

Preliminary findings of this study were presented at the 20th 19 Annual Neurocritical Care Society Meeting on 10/19/2022.

## Data Availability

The raw data supporting the conclusions of this article will be made available by the authors, without undue reservation.
